# The Regulation Mechanisms and Clinical Application of MicroRNAs in Myocardial Infarction: A Review of the Recent 5 Years

**DOI:** 10.3389/fcvm.2021.809580

**Published:** 2022-01-17

**Authors:** Chan Wu, Binghong Liu, Ruiying Wang, Gang Li

**Affiliations:** Xiamen Cardiovascular Hospital of Xiamen University, School of Medicine, Xiamen University, Xiamen, China

**Keywords:** microRNAs, myocardial infarction, mechanisms, clinical application, review

## Abstract

Myocardial infarction (MI) is the most frequent end-point of cardiovascular pathology, leading to higher mortality worldwide. Due to the particularity of the heart tissue, patients who experience ischemic infarction of the heart, still suffered irreversible damage to the heart even if the vascular reflow by treatment, and severe ones can lead to heart failure or even death. In recent years, several studies have shown that microRNAs (miRNAs), playing a regulatory role in damaged hearts, bring light for patients to alleviate MI. In this review, we summarized the effect of miRNAs on MI with some mechanisms, such as apoptosis, autophagy, proliferation, inflammatory; the regulation of miRNAs on cardiac structural changes after MI, including angiogenesis, myocardial remodeling, fibrosis; the application of miRNAs in stem cell therapy and clinical diagnosis; other non-coding RNAs related to miRNAs in MI during the past 5 years.

## Introduction

Myocardial infarction (MI) is one of the most common cardiovascular diseases globally, which can easily cause arrhythmia, ventricular remodeling, and heart failure. In 2019, millions of death patients were attributed to MI globally, which amounted to more than 20% from 2010 (1). Emerging studies demonstrated that miRNAs were critical players in MI since they participate in the genetic regulation of hundreds of essential proteins involved in signaling pathways (2–4).

MicroRNAs (miRNAs), the endogenous non-coding RNAs (ncRNAs), contain 20–24 nucleotides and bind to mRNA to block subsequent corresponding protein synthesis pathways, thereby regulating nearly all of the biological pathways (5). Studies have shown that there are at least 1,500 miRNAs in the human genome, and one miRNA can regulate multiple target genes, and the expression of one gene may also be regulated by multiple miRNAs (6, 7). This also reflects the complexity and rigor of life activities in the human body. The occurrence and development of diseases are often closely related to miRNAs. In the past decades, many scholars have proved that miRNAs as the key targets, regulated multiple cardiovascular diseases, including MI, heart failure, atherosclerosis, diabetes (8).

**Figure 1:**
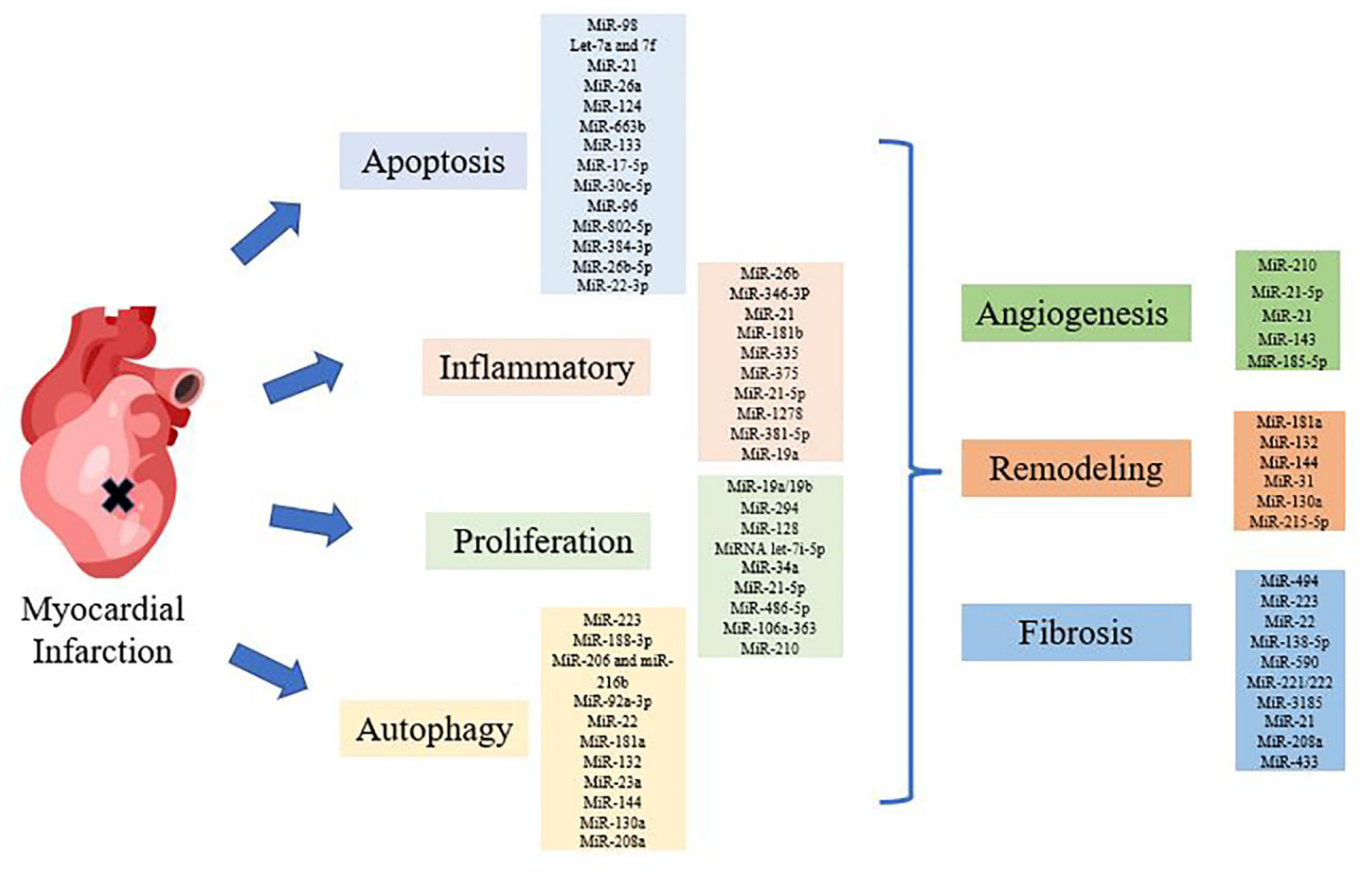
Diagram of miRNAs regulating myocardial infarction (MI). Various miRNAs regulated MI through apoptosis, inflammation, autophagy, proliferation and structural changes, such as angiogenesis, remodeling, and fibrosis.

In this review, we summarized the effect of miRNAs on MI with different mechanisms, such as apoptosis, autophagy, proliferation, inflammatory. We further summarized the regulation of miRNAs on cardiac structural changes after MI, including angiogenesis, myocardial remodeling, fibrosis. And the application of miRNAs in stem cell therapy and clinical diagnosis is introduced (Figure 1). Finally, some other ncRNAs involved in miRNA regulation of MI, such as long ncRNAs (lncRNAs), circular RNAs (circRNAs) were summarized. This study only reviewed the research on the regulation of MI by miRNAs in the past 5 years, tracing the frontier reports and time-sensitive information.

## miRNAs and Mechanism of MI

### miRNAs Regulated Cardiomyocyte Apoptosis During MI

Apoptosis is the autonomous and orderly death of cells controlled by genes to maintain the stability of the internal environment. Apoptosis can remove unnecessary or abnormal cells in the body, which has important biological significance and molecular biological mechanisms (9). Apoptosis in the cardiomyocytes occurs throughout all stages of MI. MiRNAs have emerged as potent regulators of cardiomyocyte apoptosis (7). Here we summarized the effect of miRNAs on cardiomyocyte apoptosis in MI.

The let-7 family, one of the earliest miRNAs discovered, were found to be an essential regulator of cardiomyocyte apoptosis in MI (10). The expression of miR-98, one member of the let-7 family, was reduced during MI. Further studies have found that overexpression of MiR-98 inhibited apoptosis by down-regulating Fas/Caspase3, thereby alleviating MI (11). In addition, another group showed that the expressions of let-7a and let-7f were reduced in infarcted myocardium and blood of pigs, which accelerated apoptosis, led to cardiac hypertrophy and decreased ejection fraction, and the TGFBR3 was one target of let-7 (12).

MiR-21, one of the first miRNAs discovered, regulated many signaling pathways and was regarded as an apoptotic regulator in the circulatory system (13). In H_2_O_2_-induced cardiomyocytes, miR-21 exerted its anti-apoptosis role by targeting programmed cell death 4 (PDCD4) and activator protein 1 (AP-1) pathway (14). In addition, another group discovered that miR-21 reduced inflammation, dysfunction, and myocardial remodeling after MI by targeting KBTBD7 and inhibiting the activation of p38 and NF-κB (15). However, miR-21 may be a two-sided sword. One study examined that the expression of miR-21 was up-regulated in MI mice and miR-21 regulates human peripheral blood-derived sEVs (PB-sEVs), which consisted with H9C2 cells exposed to hypoxia. And down-regulated miR-21 level by PB-sEVs improved MI mice's survival rates and cardiac function (16).

MiR-26a is downregulated in ST-elevation MI (STEMI) patients and had a strong correlation with myocardial enzyme levels. Up-regulation of miR-26a attenuated apoptosis and fibrosis and improved cardiac function by targeting ataxia–telangiectasia mutated (ATM) (17). Experiment *in vivo* and *in vitro* showed that the expression level of MiR-124 increased during MI, and down-regulating miR-124 expression reduced apoptotic cell death and improved mitochondrial dysfunction, and decreased the infarct area, as well as cardiac function in MI mice by targeting STAT3 (18). In cell-culture studies, the authors found that miR-663b inhibition reduced cardiomyocyte damage induced by hypoxia by targeting anti-apoptosis related genes (19).

The apoptosis of cardiomyocytes promotes the process of MI. We have summarized the miRNAs and their mechanisms that regulate MI through the apoptosis pathway (Table 1). MiRNA can interfere with the expression of its target genes. According to the role of the target in the process of apoptosis, the miRNA mentioned above can alleviate or aggravate myocardial infarction. MiR-663b, miR-17-5p, miR-30c-5p, miR-96, miR-802-5p, miR-384-3p, and miR-22-3p promoted MI process by targeting anti-apoptotic genes, thereby causing cardiomyocyte apoptosis (19, 24, 26, 28). Other miRNAs, such as miR-98 and miR-21, can also reduce the damage of MI by regulating different apoptotic signaling pathways (11).

**Table 1:** Summary of studies on miRNA regulating MI by apoptosis in the past 5 years.

**MiRNAs**	**Experimental Model**	**Effect in Heart or Cardiomyocyte**	**Mechanism/Target**	**References**
MiR-98	Minipigs and mice MI	Improvement	Fas-Caspase3 pathway	(11)
Let-7a and 7f	Infarcted myocardium of pigs	Improvement	Anti-apoptosis, TGFBR3	(12)
MiR-21	H_2_O_2_-induced cardiomyocytes MI mice	Improvement	PDCD4 and AP-1 pathway Targeting KBTBD7 and inhibiting the activation of p38 and NF-κB	(15)
	MI mice and hypoxia H9C2 cells		PB-sEVs	(20)
MiR-26a	Oxygen-glucose deprivation, H9c2 cells; MI in mice	Improvement	Ataxia–telangiectasia mutated	(17)
MiR-124	MI mice; H_2_O_2_-induced neonatal rat cardiomyocytes	Improvement	STAT3	(18)
MiR-663b	Hypoxia-induced H9C2 cells	Damage	BCL2L1	(19)
MiR-133	MI, rats	Improvement	Notch1 signaling pathway	(21)
MiR-17-5p	MI, mice	Damage	Tsg101	(22)
MiR-30c-5p	Coronary heart disease patients	Damage	BCL2L11	(23)
MiR-96	MI, mice	Damage	XIAP	(24)
MiR-802-5p	MI, rats	Damage	PTCH1	(25)
MiR-384-3p	MI, rats	Damage	HSP70	(26)
MiR-26b-5p	MI, mice	Improvement	Malat1	(27)
MiR-22-3p	MI, mice	Damage	NEAT1/miR-22-3p/Ltb4r1 signaling pathway	(28)

### miRNAs Regulated Myocardial Autophagy During MI

Moderate conditions of nutrient deprivation, ischemia, or hypoxia lead to autophagy slightly in the basal rate, which enables cardiomyocytes to conserve the adequate energy (29). But when myocardial ischemia is severe and lasts too long, intracellular autophagy increases sharply, which further induces cell death (30). This is also the key reason for the deterioration of the structure and function of the heart caused by autophagy (31, 32). Thus, this section will focus on the regulation of miRNAs on autophagy during MI.

Apoptosis and autophagy often occur together, and are closely related to each other in signaling pathways. MiR-223 can protect myocardial tissue from ischemic damage and myocardial cell hypoxia damage by inhibiting apoptosis and autophagy related to Akt/mTOR pathway (33). In addition, autophagy-related genes (ATGs) play an important role in regulating the occurrence and subsequent processes of autophagy (34, 35). A study revealed that miR-188-3p targeted ATG7, which led to the autophagic program and MI, while another one long non-coding RNA, AK079427, down-regulated miR-188-3p by combining with each other (36). Other research have suggested that the activities of miR-206 and miR-216b were significantly enhanced by histamine, which further inhibited autophagy. The specific regulatory mechanism was related to the inhibition of ATG13, which in turn increased the activity of the inflammatory factor caspase-8 (37). Another recent study showed that miR-92a down-regulation ameliorated endothelial cell function and alleviated MI damage by augmenting autophagy of endothelial cell via ATG4a and regulating energy metabolism of cardiac cells via Cd36 and Abca8b (38).

Generally, autophagy plays a role in protecting cells, while apoptosis is responsible for clearing aging damaged or mutated cells, thereby protecting the health of the body.

In addition to regulating apoptosis, some miRNAs can influence the process of MI by regulating autophagy (Table 2). Interestingly, miR-188-3p relieved MI by inhibiting autophagy, while miR-92a-3p aggravated MI by inhibiting autophagy (36, 38). When less affected by environmental conditions, autophagy inhibits cell apoptosis. However, in a highly stressed state, when autophagy consumes excessive amounts of intracellular proteins and organelles, making the cell unable to survive, the cell gradually develops into apoptosis. Therefore, autophagy is a double-edged sword that regulates the operation of the body.

**Table 2:** Summary of studies on miRNA regulating MI by autophagy in the past 5 years.

**MiRNAs**	**Experimental Model**	**Effect in Heart or Cardiomyocyte**	**Mechanism/Target**	**References**
MiR-223	MI, rats; neonatal rat cardiomyocytes and H9c2	Improvement	PARP-1	(33)
MiR-188-3p	Primary neonatal mice cardiomyocytes, anoxia/reoxygenation; ischemia/reperfusion, mice	Improvement	Anti-autophagy; AFP-miR-188-3p/ATG7	(36)
MiR-206 and miR-216b	Hypoxia H9c2 cardiomyocytes; MI, mice;	Improvement	Anti-autophagy: MiR-206/216b-Atg13; Atg13/FADD- caspase-8	(37)
MiR-92a-3p	MI, mice	Damage	Anti-autophagy: miR-92a/Atg4a, miR-92a/Abca8b and Cd36	(38)
MiR-22	MI, mice;	Improvement	Pro-autophagy: miR-22/PPAR-a	(39)
MiR-181a	MI, mice	Improvement	Aldo–MR pathway, miR-181a/Adamts1/Ngal	(40)
MiR-132	MI, rats	Improvement	MiR-132/IL-1β	(41)
MiR-23a	MI, rats	Damage	Inhibiting CX43 and enhancing mitophagy	(42)
MiR-144	MI, mice	Improvement	Regulating inflammation and autophagy	(43)
MiR-130a	MI, mice	Improvement	MiR-130a/PTEN/PI3K/Akt	(44)
MiR-208a	MI, rats	Damage	Pro-fibrosis; endoglin	(45)

### miRNAs Regulated Cardiomyocyte Proliferation During MI

Generally, MI patients are relieved of their dilemma through medication, intervention or surgery. However, since the cardiomyocytes in the adult mammalian heart are highly differentiated and basically do not have the ability to proliferate, pathological damage to the heart will gradually develop into irreversible damage (46). Therefore, we need effective treatments for this condition. MiRNAs have already appeared as important regulators of cardiomyocytes differentiation, proliferation, and cardiac function.

*In vivo* studies have shown that the overexpression of heart-specific miR-199a restored the proliferation and differentiation of cardiomyocytes and enhanced heart function, thereby significantly reducing the heart damage in pigs with myocardial infarction (47). The miR-17~92 cluster was a crucial driving factors of cardiomyocyte proliferation and was considered as promising targets for cardiac regeneration and repair (48). In particular, miR-19, members of the miR-17~92 cluster, reduced MI-induced cell death and preserved cardiac function by enhanced cardiomyocyte proliferation and repressed the immune in the early stage of heart attack. Therefore, miR-19 could be a candidate for valuable method for cardiovascular diseases and significantly relieve the patient's pain (49). MiR-92a, one part of the miR-17–92 cluster, is proved to be able to ameliorate the function of endothelial cell and alleviate acute MI damage (38). Studies have shown that the regenerative dysfunction of the heart of neonatal mice was often accompanied by up-regulated miR-34a, while knockout of miR-34a in the heart of adult mice restored cardiac regeneration and reduced ventricular remodeling. Further *in vitro* studies have found that miR-34a activated cell cycle activities through Cyclin D1 and Sirt1 and restored cardiomyocyte regeneration (50).

MiR-302–367 cluster is another group of miRNAs that have a regulatory effect on heart regeneration. *In vivo* studies have shown that miR-302–367 promoted the proliferation of cardiomyocytes by regulating the Hippo signaling pathway (51). Besides, miR-290–295 also played a crucial role in regulating cell cycle, DNA methylation, apoptosis, and pluripotency transcription factors. Recently, miR-294 was reported to significantly improve MI and reduce apoptosis associated with MI. The cardioprotective mechanism of miR-294 was related to multiple genes involved in the cell cycle, such as Wee1 and CyclinB1, to further restore cell cycle operation, promote angiogenesis, and maintain cardiac function (52). A study found that the heart regeneration function of neonatal mice was negatively affected by miR-128. Further research found that miR-128 inhibition in the heart of adult mice enhanced heart regeneration and improved cardiac dysfunction, which was related to the regulatory effect of miR-128 on the chromatin regulator SUZ12, further improving cell cycle activity (53).

Not only in regulating cardiomyocytes apoptosis, let-7i-5p also plays a vital role in the cardiomyocyte cell cycle and proliferation processes. Increased expression of let-7i-5p during heart growth is negatively correlated to cardiomyocyte proliferation, and knockdown of let-7i-5p enhances cardiac proliferation by regulating CCND2 and E2F2 both *in vitro* and *in vivo* (54). In addition, miR-210 restored cardiac function after MI by improving angiogenesis, cardiomyocyte proliferation, and cell survival by upregulated VEGF, Bcl-2, and β-catenin and downregulated APC, p16, and caspase-3 (55).

During MI, myocardial cells lack oxygen and energy, leading to cell death, which in turn causes cardiac dysfunction. Methods to alleviate MI include reducing the apoptosis of myocardial cells and promoting cell proliferation to restore the heart's original structure and function as much as possible. However, most mammalian cardiomyocytes have lost the ability to regenerate. Some miRNAs can regulate the cell cycle of cardiomyocytes, thereby promoting or inhibiting the proliferation of cardiomyocytes after damage (Table 3). We summarized various miRNA-related signal pathways that affect cell proliferation, including miR-294-Wee1/CyclinB-CDK1 pathway, miR-128-SUZ12-p27-Cyclin E/CDK2 pathway, miR-486-5p-MMP19-VEGFA cleavage signaling pathway, and miR302-367/Hippo signal pathway (52, 53, 57). Therefore, the research of miRNA on improving cell proliferation is very promising.

**Table 3:** Summary of studies on miRNA regulating MI by proliferation in the past 5 years.

**MiRNAs**	**Experimental Model**	**Effect in Heart or Cardiomyocyte**	**Mechanism/Target**	**References**
MiR-19a/19b	MI, mice	Improvement	Cardiomyocyte proliferation, repressing the immune response	(49)
MiR-294	MI, mice	Improvement	Cell cycle reentry, miR-294-Wee1/CyclinB-CDK1	(52)
MiR-128	MI, mice	Damage	Cell cycle reentry, miRNA-128-SUZ12-p27-CyclinE/CDK2	(53)
MiRNA let-7i-5p	Mouse ventricular cardiomyocytes, MI, mice	Damage	Cell cycle reentry, miRNA let-7i-5p/E2F2 and CCND2	(54)
MiR-34a	MI, mice	Damage	Cell cycle activity; miR-34a/Bcl2, Cyclin D1, and Sirt1	(50)
MiR-21-5p	MI, rats	Damage	Regulating cardiac microvascular endothelial cell apoptosis and angiogenesis	(56)
MiR-486-5p	MI, mice	Improvement	Regulating fibroblastic MMP19-VEGFA cleavage signaling pathway	(57)
MiR-106a-363	MI, mice	Improvement	Notch3 pathway	(58)
MiR-210	MI, mice	Improvement	Promoting cardiomyocytes proliferation, cell survival,	(55)

### miRNAs Regulated Inflammation During MI

MI (MI) initiates an inflammatory response that promotes both beneficial and harmful effects. In the first minutes after injury, pro-inflammatory cytokines in the ischemic zone synthesis and release efficiently. And the massive production of inflammatory factors affects the survival of cardiomyocytes in the infarcted area. Some miRNA control inflammation after MI to reduce apoptosis of myocytes and relieve cardiac remodeling (Table 4).

**Table 4:** Summary of studies on miRNA regulating MI by inflammation in the past 5 years.

**MiRNAs**	**Experimental Model**	**Effect in Heart or Cardiomyocyte**	**Mechanism/Target**	**References**
MiR-26b	MI, mice	Improvement	MiR-26b/PTGS2/MAPK pathway	(59)
MiR-346-3P	MI, rats	Improvement	MiR-346-3p/CaMKII	(60)
MiR-21	MI, mice	Improvement	Anti-inflammation; miR-21 /KBTBD7/p38 and NF-κB	(15)
MiR-181b	Heart failure patients	Improvement	TNF-α/IL-1/IL-6	(61)
MiR-335	MI, rats	Improvement	MAP3K2	(62)
MiR-375	MI, mice	Damage	MiR-375/PDK-1/AKT	(63)
MiR-21-5p	MI, mice	Improvement	Anti-inflammation	(64)
MiR-1278	MI, mice	Improvement	IL-22/CXCL14	(65)
MiR-381-5p	MI, mice	Improvement	CHI3L1	(66)
MiR-19a	MI, mice	Improvement	Inhibiting inflammatory cell infiltration	(67)

Ge et al. demonstrated that overexpression of miR-26b alleviated myocardial remodeling caused by MI, and prostaglandin endoperoxide synthase 2 (PTGS2) acted as a target of miR-26b to mediate the MAPK signaling pathway (59). On the contrary, some studies have shown that down-regulation of miR-26a enhanced the heart function of MI mice, which was related to the inhibitory effect of miR-26a on angiogenesis (68). Garikipati et al. found that antagonistic miR-375 exerted a protective effect on the heart by promoting angiogenesis, reducing inflammation and inhibiting cell apoptosis, which involved the PDK-1/AKT signaling pathway (63). Compared with healthy people, the expression of MiR-181b in heart failure patients decreased, while the expression of hypersensitive C-reactive protein (hsCRP) levels increased. The expression of MiR-181b in heart failure rats was negatively correlated with IL-1, TNF-α, and IL-6 (61). Wang et al. proved up-regulation of miR-335 reduced MI damage in rats by reducing oxidative stress, inflammation, and apoptosis, and MAP3K2 was an essential target for miR-335 to exert cardioprotection (62).

MI triggers an inflammatory response, which can remove debris from damaged tissues, and different inflammatory response eventually induces cardiomyocyte apoptosis. Therefore, inhibiting inflammation and reducing apoptosis are the fundamental mechanisms to alleviate the damage of cardiac structure and function after MI. Several key targets are involved in the inflammatory response to initiate or promote the inflammatory process. Some miRNAs negatively regulated the expression of inflammation-related factors, such as NF-κB, MAPK, IL-22, MAP3K2, and CHI3L1, and affected the operation of MI (Table 4). It is of great significance to study the mechanism of these miRNAs in regulating inflammation response.

## miRNAs and Heart Structural Changes After MI

### The Effect of miRNAs on Angiogenesis After MI

In addition to large blood vessels, obstruction of microvessels can also cause or accelerate cardiovascular diseases, so angiogenesis has great potential in the treatment of ischemic diseases (69). The miRNAs related to angiogenesis potential on ischemic cardiovascular diseases are summarized following (Table 5).

**Table 5:** Summary of studies on miRNA regulating angiogenesis, fibrosis, and myocardial remodeling after MI in the past 5 years.

**Classification**	**MiRNAs**	**Experimental Model**	**Effect in Heart or Cardiomyocyte**	**Mechanism**	**Reference**
Angiogenesis	MiR-210	MI, mice	Improvement	Pro-angiogenesis; APC; miR-210/Efna3	(55, 70)
	MiR-21-5p	MI, mice	Improvement	Phosphatase and tension homolog/Akt pathway;	(71)
	MiR-21	MI, mice	Damage	Nitrogen metabolism, miR-21/STRN/NOS3	(20)
	MiR-143	MI patient serum	Damage	MiR-143/IGF-IR/NO	(72)
	MiR-185-5p	MI, mice	Damage	MiR-185-5p/cathepsin K	(73)
Fibrosis	MiR-494	MI, rat	Improvement	MiR-494/LRG1/Wnt	(74)
	MiR-223	MI, rat; cardiac fibroblasts, transfection	Damage	Pro-fibrosis; miR-223/RASA1	(75)
	MiR-22	MI, mice; rat cardiac fibroblast, transfection	Damage	Pro-fibrosis; miR-22 /caveolin-3/pPKCε	(39)
	MiR-138-5p	MI, mice	Improvement	MiR-138-5p/RhoC axis	(76)
	MiR-590	MI, rat	Improvement	TGF-β1	(77)
	MiR-221/222	MI, mice	Improvement	MiR-221/222/p38/NF-κB pathway	(78)
	MiR-3185	MI, mice	Damage	Inhibiting the upregulation of Col I and Col III induced by TGF-β1	(79)
	MiR-21	MI, mice; cardiac fibroblast, transfection	Damage	Pro-fibrosis; TGF-β/Smad7 signaling pathway	(80)
	MiR-208a	MI, rat	Improvement	MiR-208a/endoglin	(45)
	MiR-433	MI, mice; cardiac fibroblast, transfection	Damage	Pro-fibrosis; miR-433/AZIN1/JNK1/TGFβ-Smad3	(81)
Myocardial remodeling	MiR-181a	MI, mice;	Improvement	Against cardiac remodeling: miR-181a/Adamts1/Ngal	(40)
	MiR-132	MI, rat	Improvement	Anti-apoptosis, miR-132/IL-1β	(41)
	MiR-144	MI, mice	Improvement	Against cardiac remodeling	(43)
	MiR-31	MI, rat	Damage	Post-ischemic adverse remodeling	(82)
	MiR-130a	MI, mice	Improvement	MiR-130a/PTEN/PI3K/Akt	(44)
	MiR-215-5p	MI, rat	Improvement	MiR-215-5p/ZEB2 Axis	(83)

MiR-21, one of the first miRNAs discovered, regulates many signaling pathways and plays a vital role in the cardiovascular system (13). A study shows that miR-21-5p is a component of exosomes, which can enhance angiogenesis and cardiomyocyte survival through augmented phosphorylation of Akt via the inhibition of PTEN in the recipient cells (71). Extracellular vesicles (EVs)/exosomes, which are responsible for cell-cell information transmission (84), can mediate the cardioprotective effects of stem cells through paracrine action (85, 86). The study showed exosomes derived from serum of MI patients accelerated cardiac angiogenesis. Furthermore, microarray assays demonstrated that the regulation of exosomes on cardiac angiogenesis was related to the inhibitory effect of miR-143, which bind to IGF-IR and enhanced the production of NO (72). The function of endothelial cells plays an indispensable role in the process of MI. In human umbilical vein endothelial cells (HUVECs), miR-185-5p down-regulates the expression of cathepsin K, thereby hindering angiogenesis, leading to further impairment of cardiac function in MI mice (73).

During MI, blood vessel damage of the heart accelerates energy metabolism disorder, so vascular regeneration is significant. MiR-210 and miR-21-5p promoted vascular regeneration through different pathways, while miR-143 and miR-185-5p blocked cardiac angiogenesis and aggravated heart damage (Table 5). Therefore, it is necessary to conduct in-depth research on the mechanism of miRNA regulating vascular regeneration after MI.

### The Effect of miRNAs on Fibrosis After MI

Even after interventional therapy opens MI patients to block the blood vessels, which greatly reduces the mortality of MI patients, the development of cardiac injury to myocardial fibrosis in MI patients cannot be curbed. Myocardial fibrosis can also be regarded as an important signal for heart remodeling, which further leads to arrhythmia, cardiac dysfunction and even heart failure (87, 88). So, the study of myocardial fibrosis has predominant clinical significance, and miRNAs may become promising candidates for regulating myocardial fibrosis.

The study reported that increased miR-22 levels promoted fibrosis process by enlarged proliferation of cardiac fibroblasts. In addition, others reported that in MI mice models and cardiac fibroblasts, miR-21 promoted cardiac fibrosis via the TGF-β/Smad7 signaling pathway (80). The research showed that miR-494 targeted and downregulated leucine-richalpha-2-glycoprotein 1(LRG1), resulting in the inactivation of the Wnt signaling pathway and promoting proliferation and migration and invasion ability of fibroblasts and VECs (74). Inhibition of miR-223, as a pro-fibrosis factor, can accelerate the transformation of cardiac fibroblasts into myofibroblasts through the RASA1/TGF-β1 signaling pathway (75). In contrast, the downregulation of miR-22 level reduced cardiac fibroblasts via miR-22/Cav3/p-PKCε signaling (39). Decreased levels of miR-22 in MI elderly mice significantly enhanced heart function and prevented ventricular remodeling by promoting autophagy activity. However, the regulatory effect of miR-22 on young mice was not significant. Clinical related studies have also shown that the level of miR-22 in patients with heart failure was positively correlated with patient mortality (89). Tao et al. reported that elevated levels of miR-433 also appeared in three cardiovascular disease models with fibrotic transition trends, and especially expressed in fibroblasts in heart tissue. In comparison, inhibition of miR-433 attenuates cardiac fibroblast proliferation and myofibroblast differentiation *in vitro*. Mechanistically, mitogen-activated protein kinase 8 (JNK1) and antizyme inhibitor 1 (AZIN1) were determined as important targets of miR-433 (81). A previous study reported miR-208 is a marker of myocardial necrosis (90). In another research, Shyu et al. demonstrated that antagonistic miR-208a prevented myocardial fibrosis after MI via endoglin (45).

There are also reports that miR-138-5p, miR-590, and miR-221/222 inhibited fibrosis after MI through RhoC, TGF-β1, and p38/NF-κB, respectively. And miR-3185 promoted myocardial fibrosis by increasing collagen deposition. The above results indicated that different miRNAs targeted multiple mRNAs to encourage or inhibit myocardial fibrosis after MI (Table 5).

### The Effect of miRNAs on Myocardial Remodeling After MI

Despite stent technology, procedural techniques, and adjunctive pharmacotherapy reduced death rate of MI, survivors of MI also experienced ventricular remodeling and even heart failure (HF) (91). Cardiac remodeling is a process that involves cellular, molecular, and physiological changes, including inflammation, fibrosis, and ventricular dysfunction and is an important factor affecting the occurrence of cardiovascular events, long-term survival rate, and quality of life (92).

The occurrence and development of cardiac remodeling involved a variety of miRNAs. This section focuses on summarizing the role of miRNAs in cardiac remodeling caused by MI (Table 5). MiR-181a, upregulated in MI, is an important regulatory factor of the aldosterone–mineralocorticoid receptor (Aldo–MR) pathway, which drive cardiac arterial stiffness, fibrosis, hypertrophy, and cardiac remodeling by promoting inflammation and oxidative stress, which were involved in its downstream factors such as NGAL (lipocalin-2) (40, 93, 94). Interestingly, other miRNAs derived from the miR-181 family have differential effects on heart function regulation. Study showed that miR-181b-5p has a deleterious cardiac impact and can decrease MI damage via PI3K/Akt signaling pathway (95). Another study shows that miR-132 inhibition was meaningful for suppressing cardiac remodeling by regulating IL-1β-related apoptosis (41). Pan et al. reported the restoration of miR-101a improves cardiac function in rats after MI by inhibiting interstitial fibrosis (96). In another study, the researchers demonstrated that in mice's MI (MI) model, the level of miR-144 decreased, which contributes to the LV remodeling via modification of local inflammatory and auto-phagocytic pathways (43). Martinez et al. revealed that the time-dependent upregulation of miR-31 plays a significant role in adverse remodeling following rat MI, inhibition of miR-31 modulates several genes, such as Tnnt2, Timp4, and E2f6, which is vital to the recovery of cardiac function and structure after MI (82). Besides, Lu et al. showed that miR-130a remarkably enhanced cardiac function and prevented remodeling after MI by activating PTEN/PI3K/Akt signaling pathway (44).

Myocardial remodeling manifests ventricular cavity enlargement, progressive hypofunction, collagen deposition outside myocardial cells, inflammatory cell infiltration, and apoptosis. Some miRNAs, for example, miR-181, miR-144, miR-130a, regulate phenotypes related to ventricular remodeling by down-regulating the expression of related genes (40, 43, 44), which can be regarded as potential targets for anti-ventricular remodeling after MI (Table 5).

## The Involvement of miRNAs in the Diagnosis and Treatment of MI

### Stem Cell Therapy on MI

External stimulation triggers the myocardial self-defense system, which reduces the loss of myocardial cells to a certain extent and increases the proliferation of myocardial cells, but it is not enough to achieve cardiac repair (85, 97, 98). Cell therapy is to achieve tissue repair by transplanting stem cells or their derived cells into damaged tissues, which has gradually become an alternative therapy that has attracted much attention (Table 6) (109, 110).

**Table 6:** Summary of studies on miRNA's involvement in the diagnosis and treatment of MI in the past 5 years.

**Classification**	**MiRNAs**	**Experimental Model**	**Effect in Heart or Cardiomyocyte**	**Mechanism**	**References**
Stem cells	MiR-25-3p	Oxygen glucose deprivation, adult mice cardiomyocytes; ischemia-reperfusion, mouse	Improvement	Anti-apoptosis, MSCs-Exo/miR-25-FASL/PTEN; anti-inflammation, MSCs-Exo/miR-25-EZH2-eNOS, SOCS3	(99)
	MiR-497	Oxygen glucose deprivation, neonatal rat cardiomyocytes; MI, mice	Improvement	hCVPC-EVs/MALAT1/miR-497	(100)
	MiR-185	MI, mice	Improvement	BMSCs-Exo/miR-185/SOCS2	(101)
	MiR-30e	MI, rats	Improvement	NF-κB p65/Caspase-9 signaling	(102)
	Mir-125b	Oxygen glucose deprivation, neonatal mouse cardiomyocytes; MI, mouse	Improvement	MSCs-Exo/miR-125b/p53-Bnip3	(103)
	MiR-495	MI, mice	Damage	hiPSCs angiogenesis; miR-495/VEZF1	(104)
	MiR-210	MI, mouse	Improvement	Angiogenesis; MSCs-EVs/miR-210/Efna3	(55, 70)
Clinical application	MiR-320a	Patients with STEMI, the atrium of ischemic patients with heart failure	Biomarkers positively associated with LVAR in STEMI	Unknown	(105)
	MiR-1 and miR-499	Patients with AMI or SCD	Discriminating SCD from AMI	Unknown	(90)
	MiR-208	Patients with AMI	Distinguishing AMI from control cases	Unknown	(45)
	MiR-331 and miR-151-3p	Patients with STEMI	Biomarkers associated with plaque rupture	It may be in the vulnerable plaque	(106)
	MiR-26b-5p, miR-660-5p, and miR-320a	Patients with STEMI	Biomarkers of adverse cardiovascular events	Regulating apoptosis, cardiac remodeling, platelet production	(107)
	MiR-133b and miR-21	patients with CAD	Biomarkers of prediction and diagnosis	Unknown	(108)

Mesenchymal stem cells (MSCs) in multiple MI models have been shown to inhibit apoptosis, regenerative, and inflammatory (111, 112), which was involved in over 50 miRNAs in MSC-derived exosomes (113). MiR-25-3p, one of the exosomal miRNA from MSCs, decreased the level of apoptosis directly by targeting FASL and PTEN (114). The other mechanism is that MiR-25-3p reduced the expression of EZH2 and H3K27, which led to the anti-inflammatory gene SOCS3 promoter was decreased (99), leading to SOCS3 restoration and decreased proinflammatory cytokines (115). Thus, exosomes carrying miR-25-3p may become a promising way to treat MI.

MSCs are derived from mesenchymal tissues like bone marrow (BMSC), which are stromal cells with proliferation function (116). The expression level of MiR-185, bone marrow mesenchymal stem cells-derived exosomal (BMSCs-Exo), reduced in the mouse heart tissues after MI. BMSCs-Exo containing miR-185 in MI mice exerted cardiac remolding inhibitory effect by targeting suppressors of cytokine signaling 2 (SOCS2) (101). In addition, another study reported that MSCs treatment recovered damage to the structure and function of the heart in MI by reducing apoptosis and autophagy via miR-125-5p/p53/Bnip3 signaling pathway (103). As a paracrine product of MSC, MSC-EVs play a role in promoting vascular regeneration and functional recovery in the damaged heart. Further studies have shown that the angiogenesis effect of MSC-EVs was highly related to miR-210/Efna3 (70). Human pluripotent stem cells derived from cardiovascular progenitor cells are regarded as a valued method for myocardial repair (110, 117). A study *in vivo* has reported injection of human cardiovascular progenitor cells extracellular vesicles into infracted myocardial tissue enhanced cardiac function, accelerated vascularization and prevented fibrosis via targeting miR-497 (100). In another study, Liang et al. revealed that miR-495 represented a new target for improving EC generation from human induced pluripotent stem cells (hiPSCs) and promoting angiogenesis and transplant of hiPSC-derived ECs in infarcted heart. The cardiac regulatory mechanism was that miR-495 affected EC-related gene expression by targeting VEZF1 (104).

### Clinical Application of MI

A clinical study on the analysis of miRNA arrays in humans was used to screen out human heart-specific miRNAs for the diagnosis of heart-related diseases. The results showed that miR-499, which was mainly enriched in the heart, was highly expressed in the plasma of patients with acute MI (118). In the other study, the investigator found miR-499 and miR-1 were the most sensitive markers in discriminating sudden cardiac death (SCD) from acute MI, whereas miR-208 showed the highest specificity in distinguishing acute MI from control cases (90). A multicenter prospective study showed that miR-320a, miR-660-5p, and miR-26b-5p to be highly correlated with patients with ST-segment-elevation MI (STEMI), and these three miRNAs might be further used for the improvement of risk assessment and follow-up intervention in STEMI patients (Table 6). Significantly, apoptosis, ventricular remodeling, and platelet production were primary regulatory mechanism of these miRNAs (107).

The formation of atherosclerotic plaque has the risk of causing occlusion of arteries and blood vessels. Once the vulnerable plaque ruptures, thrombus will form, which can further develop into ischemic infarction, stroke and other complications (119, 120). A study design contributes to screening out differential miRNAs in acute coronary artery occlusion due to atherosclerotic plaque rupture. The results suggest that Elevated miR-151-3p and miR-331 appeared even earlier than myocardial infarction markers, indicating that these two miRNAs have higher sensitivity to atherosclerotic plaque rupture (106). Atherosclerosis is regarded as a pathological change in the early stage of coronary syndrome, and it is difficult to be detected in the early stage. Therefore, there is still a lack of fast and effective methods for early detection of atherosclerosis. In a clinical study, investigators recruited 147 subjects, including 78 topics with angiographically proven coronary artery disease (CAD), 15 normal coronary arteries issues with atherosclerosis, and 54 healthy individuals. They found miR-133b was decreased in plasma of CAD patients, whereas miR-21 was elevated. Notably, both of these miRNAs have a significant correlation with the deterioration of heart disease (108).

Another study about circulating miRNA-1 (miR-1) in acute MI (AMI) diagnosis and prognosis. Another clinical trial recruited 337 patients with acute chest pain, including 174 patients with acute MI and 163 patients with non-acute MI. The results of the study showed that miR-1 was significantly different between the two groups. The miR-1 in the plasma samples of patients with acute MI was significantly increased, indicating that the diagnosis results of miR-1 and the myocardial infarction marker cTnI were similar. In addition, miR-1 combined with CK-MB, cTnI, and other clinical and laboratory indicators (including BMI, HR, TG, and LDL-C, smoking history, and renal dysfunction) had the highest diagnostic efficiency (121). Other investigators revealed that circulating miR-1 and miR-133b, cardiac-specific miRNA, may as early alternative markers for STEMI patients (122). Another clinical study of 20 STEMI patients found that after the coronary blood flow was restored, the levels of miR-1 and miR-133b increased rapidly either alone or at the same time, and the increase of miR-1 was associated with left ventricular dysfunction (123). MiR-320a was determined inpatient with STEMI and positively associated with left ventricular adverse remodeling (LVAR) (105).

The clinical diagnosis of MI is mainly based on the patient's clinical symptoms, abnormalities in the electrocardiogram, and specific biomarkers. Among them, cTnT and cTnI are currently the preferred MI markers. However, due to the relatively delayed release of troponin, finding new biomarkers is incredibly beneficial to the early diagnosis of MI. According to the above introduction, miRNA can be detected more sensitively and quickly and used as a potential early diagnosis method for MI.

## The Regulation of Other ncRNAs on miRNAs During MI

In addition to miRNAs, there are some other ncRNAs, such as lncRNAs and circRNAs, which also play an important role in the occurrence and development of MI (124). A further summary found that lncRNAs and circRNA acted as sponges of miRNAs, and regulated MI by affecting the function of miRNAs (125).

### LncRNAs as Sponges of miRNAs

LncRNAs are composed of more than 200 nucleotides and involved in cell growth, differentiation, proliferation and other processes (126). During MI, some lncRNAs can interact with miRNAs to regulate the target molecules corresponding to miRNAs (Table 7). We have previously summarized that miR-26a reduced MI through regulation of apoptosis and inflammation (17). Su et al. found that lncRNA MIRF acted on the sponge of miR-26a and reduces apoptosis during MI by regulating the pro-apoptotic protein Bak1 (139). In addition, there is also a report proving that autophagy was a key mechanism after the interaction between lncRNA MIRF and miR-26a (127). Notably, lncRNA MALAT1 acted on miR-320 to aggravate cell apoptosis (130), while the interaction between lncRNA MALAT1 and miR-26b-5p promoted microcirculation repair after MI (27). These studies showed that lncRNA MALAT1 acted on different miRNAs to have different effects on MI. Otherwise, lncRNA GAS5 acted as the sponges of miR-21, miR-525-5p, and miR-142-5p, which can aggravate MI through different mechanisms (131–133). In summary, one type of lncRNA, as the sponges for multiple miRNAs, inhibited the function of miRNAs, and then exerted different physiological and pathological regulatory effects.

**Table 7:** Summary of studies on lncRNAs as sponges of miRNA during MI in the past 5 years.

**MiRNAs**	**LncRNAs**	**Experimental Model**	**Effect of lncRNA in MI**	**Mechanism/Target**	**References**
MiR-26a	Mirf	MI mice	Improvement	Autophagy	(127)
MiR-7-5p	ANRIL	Hypoxia-induced H9C2 cells	Improvement	SIRT1	(128)
MiR-143	UCA1	MI rats	Improvement	MiR-143/MDM2/p53 axis	(129)
MiR-320	MALAT1	MI mice	Damage	Apoptosis	(130)
MiR-26b-5p	MALAT1	MI mice	Improvement	Mitochondrial dynamics	(27)
MiR-21	GAS5	MI rats	Damage	PDCD4/PI3K/AKT	(131)
MiR-525-5p	GAS5	Hypoxia-induced H9C2 cells	Damage	CALM2	(132)
MiR-142-5p	GAS5	Hypoxia-induced H9C2 cells	Damage	TP53INP1	(133)
MiR-378	PCFL	MI mice	Damage	Fibrosis	(134)
MiR-101a-3p	XIST	Hypoxia-induced neonatal mice cardiomyocytes	Damage	FOS	(135)
MiR-34-5p	SNHG7	MI mice	Damage	ROCK1	(136)
MiR-519d-3p	HOTAIR	MI rats	Improvement	Apoptosis	(137)
MiR-132-3p	TUG1	H_2_O_2_-induced primary cardiomyocytes	Damage	HDAC3	(138)

### CircRNAs as Sponges of miRNAs

Similar to lncRNAs, circRNAs also acted as the sponges for miRNAs during MI, thereby reducing/aggravating MI (Table 8). Studies have shown that circRNA MFACR inhibited the protective effect of miR-652-3p on myocardial mitochondrial function in MI mice (140). At the same time, circRNA MFACR also promoted cell apoptosis in MI region by binding miR-125b (141). In addition, circRNA POSTN, as a sponge of miR-96-5p, led to increased myocardial infarction area and decreased cardiac function by targeting BNIP3 (142). Some circRNAs can aggravate MI, such as ACAP2, MAT2B, Cdr1as, but there are also some circRNAs that improve MI injury (143–145). Zhu et al. found that circRNA SNRK targeting miR-103-3p reduced apoptosis and promoted proliferation, thereby improving cardiac function, through the GSK3β/β-catenin pathway (146). Studies have also shown that overexpression of circRNA Hipk3 improved endothelial cell function, promoted angiogenesis, and reduced MI damage through the Notch1/miR-133a axis (147). In short, the regulatory effect of circRNAs on MI mainly depends on the regulatory mechanism of miRNAs' targets.

**Table 8:** Summary of studies on circular RNAs as sponges of miRNA during MI in the past 5 years.

**MiRNAs**	**CircRNA**	**Experimental Model**	**Effect of circular RNA in MI**	**Mechanism/Target**	**References**
MiR-96-5p	POSTN	MI mice	Damage	BNIP3	(142)
MiR-125b	MFACR	MI patients	Damage	Apoptosis	(141)
MiR-652-3p	MFACR	MI mice	Damage	MTP18	(140)
MiR-532	ACAP2	MI patients	Damage	Apoptosis	(143)
MiR-103-3p	SNRK	MI rats	Improvement	GSK3β/β-catenin pathway	(146)
MiR-133a	Hipk3	MI mice	Improvement	Notch1	(147)
miR-133	MAT2B	Hypoxia-induced H9C2 cells	Damage	Inflammation	(144)
Let-7a-5p	101237	Hypoxia-induced primary cardiomyocytes	Damage	Apoptosis and autophagy	(151)
MiR-15b	Ttc3	MI rats	Improvement	Apoptosis	(152)
MiR-7a	Cdr1as	MI mice	Damage	PARP and SP1	(145)

## Conclusions

MiRNAs are highly conservative in evolution, with tissue specificity and timing in expression, so they play an important role in various life activities of organisms. During the pathogenesis of MI, the corresponding miRNA changes and regulates the processes of cardiomyocyte apoptosis, myocardial fibrosis, angiogenesis, and inflammation after MI. Therefore, the identification of miRNA related to MI is crucial for developing miRNA diagnosis and treatment methods, and the corresponding clinical research is also ongoing.

This study concluded that miRNAs could reduce or aggravate MI by regulating apoptosis, autophagy, cell proliferation, and inflammation. MiRNAs can also regulate myocardial fibrosis, cardiac vascular regeneration, and ventricular remodeling after MI. The specific mechanism of miRNAs regulating MI was introduced. At present, the mechanisms for miRNAs to regulate MI were also very different. Some miRNAs may affect the process of MI through a variety of signaling pathways. MiR-21 can inhibit apoptosis and inflammation by targeting KBTBD7, regulate angiogenesis through STRN/NOS3, and promote myocardial fibrosis through the TGF-β/Smad7 signaling pathway. This also shows that miRNAs can target a variety of mRNAs, affecting the characteristics of downstream pathways. In addition, some MiRNAs, such as miR-320a and miR-208, can also be used as potential biomarkers for the diagnosis of MI. In addition to the above mechanisms, abnormal lipid metabolism is involved in the occurrence and development of atherosclerosis, and is also the inducement and main driver of MI (148). Current studies have shown that miRNAs regulated lipid metabolism through lipoprotein synthesis and reverse cholesterol transport (149). However, there are few reports related to MI, and the knowledge system needs to be further expanded (150). In fact, the regulation of MI by miRNAs is also affected by other ncRNAs, such as lncRNAs, circRNAs, which change the regulation of miRNAs on downstream pathways by binding to the sponge region of miRNAs. Therefore, miRNAs are potential for basic research and clinical development of MI.

Although miRNAs have made some progress in studying the mechanism of the occurrence and development of MI, there are still some limitations that need to be further investigated. For example, the specific regulation pathways of most miRNAs *in vivo* are not yet clear; most of the current researches are limited to animal experiments, and a large number of clinical trials are needed for verification; although different methods can knock down miRNA, most miRNA overexpression still requires viral transfection; the off-target effect of miRNA hinders potential transformation studies in humans. But it is worthy of recognition that the discovery of miRNAs opened up a new path for MI research. With the advancement of science and technology and the research of countless researchers, I believe that these problems will be overcome one by one. Thus, more efforts are required to deepen our understanding of the role of miRNA networks in MI. This will be particularly important when seeking safe and effective diagnosis and treatment.

## Author Contributions

GL and RW designed experiments. CW and BL carried out investigations. CW and RW wrote the manuscript. GL supervised the entire study, revised the manuscript, and provided the funding. All authors contributed to the writing and editing of the manuscript.

## Funding

This study was supported by a Joint Fund for Science and Technology Cooperation across the Taiwan Straits from the National Natural Science Foundation and Fujian Province, China (Grant No. U1605226), a Science and Technology Project from Xiamen Science and Technology Bureau, Fujian Province, China (Grant Nos. 3502Z20184025 and 3502Z20184024).

## Conflict of Interest

The authors declare that the research was conducted in the absence of any commercial or financial relationships that could be construed as a potential conflict of interest.

## Publisher's Note

All claims expressed in this article are solely those of the authors and do not necessarily represent those of their affiliated organizations, or those of the publisher, the editors and the reviewers. Any product that may be evaluated in this article, or claim that may be made by its manufacturer, is not guaranteed or endorsed by the publisher.
