# Corrigendum: The Regulation Mechanisms and Clinical Application of MicroRNAs in Myocardial Infarction: A Review of the Recent 5 Years

**DOI:** 10.3389/fcvm.2022.900621

**Published:** 2022-05-12

**Authors:** Chan Wu, Binghong Liu, Ruiying Wang, Gang Li

**Affiliations:** Xiamen Cardiovascular Hospital of Xiamen University, School of Medicine, Xiamen University, Xiamen, China

**Keywords:** microRNAs, myocardial infarction, mechanisms, clinical application, review

In the published article, there was an error in affiliation 1. Instead of “School of Medicine, Cardiovascular Hospital of Xiamen University, Xiamen University, Xiamen, China,” it should be “Xiamen Cardiovascular Hospital of Xiamen University, School of Medicine, Xiamen University, Xiamen, China.”

The authors apologize for this error and state that this does not change the scientific conclusions of the article in any way. The original article has been updated.

## Publisher's Note

All claims expressed in this article are solely those of the authors and do not necessarily represent those of their affiliated organizations, or those of the publisher, the editors and the reviewers. Any product that may be evaluated in this article, or claim that may be made by its manufacturer, is not guaranteed or endorsed by the publisher.

